# Isolation of *Pseudomonas aromaticivorans* sp. nov from a hydrocarbon-contaminated groundwater capable of degrading benzene-, toluene-, *m*- and *p*-xylene under microaerobic conditions

**DOI:** 10.3389/fmicb.2022.929128

**Published:** 2022-09-20

**Authors:** Sinchan Banerjee, Anna Bedics, Erika Tóth, Balázs Kriszt, André R. Soares, Károly Bóka, András Táncsics

**Affiliations:** ^1^Department of Molecular Ecology, Institute of Aquaculture and Environmental Safety, Hungarian University of Agriculture and Life Sciences, Gödöllő, Hungary; ^2^Department of Microbiology, Eötvös Loránd University, Budapest, Hungary; ^3^Department of Environmental Safety, Institute of Aquaculture and Environmental Safety, Hungarian University of Agriculture and Life Sciences, Gödöllő, Hungary; ^4^Group for Aquatic Microbial Ecology, Institute for Environmental Microbiology and Biotechnology, University of Duisburg-Essen, Essen, Germany; ^5^Department of Plant Anatomy, Eötvös Loránd University, Budapest, Hungary

**Keywords:** biodegradation, xylene, BTEX, *Pseudomona*, groundwater

## Abstract

Members of the genus *Pseudomonas* are known to be widespread in hydrocarbon contaminated environments because of their remarkable ability to degrade a variety of petroleum hydrocarbons, including BTEX (benzene, toluene, ethylbenzene and xylene) compounds. During an enrichment investigation which aimed to study microaerobic xylene degradation in a legacy petroleum hydrocarbon-contaminated groundwater, a novel Gram-stain-negative, aerobic, motile and rod-shaped bacterial strain, designated as MAP12^T^ was isolated. It was capable of degrading benzene, toluene, *meta*- and *para*- xylene effectively under both aerobic and microaerobic conditions. The 16S rRNA gene sequence analysis revealed that strain MAP12^T^ belongs to the genus *Pseudomonas*, with the highest 16S rRNA gene similarity to *Pseudomonas linyingensis* LYBRD3-7*^T^* (98.42%), followed by *Pseudomonas sagittaria* JCM 18195*^T^* (98.29%) and *Pseudomonas alcaliphila* JCM 10630*^T^* (98.08%). Phylogenomic tree constructed using a concatenated alignment of 92 core genes indicated that strain MAP12^T^ is distinct from any known *Pseudomonas* species. The draft genome sequence of strain MAP12^T^ is 4.36 Mb long, and the G+C content of MAP12^T^ genome is 65.8%. Orthologous average nucleotide identity (OrthoANI) and digital DNA–DNA hybridization (dDDH) analyses confirmed that strain MAP12^T^ is distinctly separated from its closest neighbors (OrthoANI < 89 %; dDDH < 36%). Though several members of the genus *Pseudomonas* are well known for their aerobic BTEX degradation capability, this is the first report of a novel *Pseudomonas* species capable of degrading xylene under microaerobic conditions. By applying genome-resolved metagenomics, we were able to partially reconstruct the genome of strain MAP12*^T^* from metagenomics sequence data and showed that strain MAP12*^T^* was an abundant member of the xylene-degrading bacterial community under microaerobic conditions. Strain MAP12^T^ contains ubiquinone 9 (Q9) as the major respiratory quinone and diphosphatidylglycerol, phosphatidylglycerol, phosphatidylethanolamine as major polar lipids. The major cellular fatty acids of strain MAP12^T^ are summed feature 3 (C_16:1_ω6c and/or C_16:1_ω7c), C_16:0_ and summed feature 8 (C_18:1_ω6c and/or C_18:1_ω7c). The results of this polyphasic study support that strain MAP12^T^ represents a novel species of the genus *Pseudomonas*, hence the name of *Pseudomonas aromaticivorans* sp. nov. is proposed for this strain considering its aromatic hydrocarbon degradation capability. The type strain is MAP12^T^ (=LMG 32466, =NCAIM B.02668).

## Introduction

Benzene, toluene, ethylbenzene and xylene (BTEX) are a group of major organic pollutants with crucial public health concerns due to their genotoxic and carcinogenic properties (Díaz, 2004). They are considered an environmental threat due to their relatively high-water solubility and stability against degradation. Despite its well-known toxicity, this group of aromatic compounds has been used extensively in industries, and thus found as a ubiquitous pollutant, making it a significant risk to groundwater resources. Degradation of these contaminants occurs in the environment naturally by indigenous microorganisms that feed on these contaminants as carbon and energy source (Xu et al., 2018).

Rapid aerobic degradation of BTEX-compounds leads to a scarcity of oxygen in contaminated subsurface environments, resulting in the formation of hypoxic, and eventually anaerobic conditions. Contaminant removal from these environments may be aided by microorganisms and can effectively biodegrade aromatic compounds microaerobically. Various bacteria have been reported to degrade BTEX-compounds (Prince et al., 2019). Among them, *Pseudomonas* species are often reported as major players in such contaminated sites that play a significant role in the degradation of BTEX (Ridgway et al., 1990; Brusa et al., 2001; Yu et al., 2001; Jahn et al., 2005). Though the bioremediation potential of genus *Pseudomonas* has been described in several reports, according to literature, to date, there is only limited information on *Pseudomonas* spp. that can degrade benzene and xylene under microaerobic conditions (Mahendran et al., 2006). Moreover, only a handful of other bacterial strains are known to degrade benzene and *para*-xylene in oxygen-limited environments, and as a consequence they are the least degradable and most persistent BTEX-compounds in subsurface ecosystems (Margesin et al., 2003; Wartell et al., 2021).

Genus *Pseudomonas* belongs to the family Pseudomonadaceae within γ-Proteobacteria, originally first described by Migula (1894). *Pseudomonas* is a species-rich genus. At the time of writing, there have been more than 200 species with valid names within this genus (LPSN^1^) (Parte et al., 2020). Members of this genus are Gram-stain-negative, non-spore-forming, catalase and oxidase test positive (Lin et al., 2013), rod-shaped bacteria containing one or several polar flagella or peritrichous flagella. The genus *Pseudomonas* is characterized by the presence of ubiquinone 9 as the major respiratory quinone (Jia et al., 2020; Wang et al., 2020). The genome G+C content values vary between 58 and 69 mol% (Lang et al., 2010). *Pseudomonas* spp. have a high degree of physiological adaptability since they can grow on both simple and complex organic carbon compounds at 4–42°C and pH 4.0–8.0 (Moore et al., 2006). Owing to diverse metabolism, members of this genus are able to survive in a wide range of niches like plants, animals, soil, water, and air (Gupta et al., 2008), including soils containing high levels of pollutants, such as xenobiotics (Manickam et al., 2008) e.g., haloaromatic-compounds (Stolz et al., 2007), alkylphenols, and petroleum hydrocarbons (Hirota et al., 2011).

During investigation of microaerobic xylene-degrading bacterial community diversity of groundwater of a decade-old petroleum hydrocarbon-contaminated site at Siklos, Hungary (Táncsics et al., 2013), by using selective enrichment method, strain MAP12^T^ was isolated and proved to be a yet undescribed lineage of the genus *Pseudomonas*. The Siklos site is an extensively studied, legacy hydrocarbon contaminated site of Hungary, which is predominantly contaminated by xylene (Banerjee et al., 2022). The aim of this present study was to clarify the taxonomic status of this presumably novel member of the genus *Pseudomonas* using a polyphasic taxonomic approach, including whole genome sequencing, physiological, biochemical, and chemotaxonomic analyses. Along with the evaluation of its capability as a promising candidate for aromatic hydrocarbon degradation under microaerobic conditions and deep dive into its genome features to understand BTEX degradation associated genomic details.

## Materials and methods

### Enrichment process and bacterial strain isolation

To enrich and isolate indigenous microaerobic xylene-degrading bacterial strains, triplicate microaerobic (≤0.5 mg/L dissolved O2) enrichments were established in 100 mL crimp top serum bottles containing 45 mL of vitamin supplemented mineral salt medium (MSV medium) (Révész et al., 2020) added with 1 mM xylene mixture, i.e., *ortho*-, *meta*- and *para*-xylene mixture (1:1:1) as a sole source of carbon and energy and 5 mL of contaminated groundwater sample to enrich xylene degraders selectively. Groundwater sample was obtained from a gasoline contaminated site of Siklós, Hungary (Táncsics et al., 2012) from the monitoring well No. ST2 situated at the center of the contaminated zone. The sample was collected into sterile 1-L serum bottles, keeping no headspace, and transported to the laboratory, at 4°C in the dark for further investigation. Prior to the inoculation, microaerobic microcosms were sparged with N_2_/CO_2_ (80:20, v/v) for 10 min. After that, sterile air (0.2 μm pore size filtered) was injected via the butyl-rubber septa to achieve the desired dissolved oxygen concentration. To ensure the prevalence of microaerobic conditions, the bottles were monitored non-invasively by Fibox 3 trace v3 fiber optic oxygen meter with PSt3 sensor spots (PreSens) at regular intervals and oxygen was continuously replenished in the enrichments whenever needed. Microcosms were incubated in a shaker incubator (28°C, 150 rpm) for a week. Then 5 mL inoculum from each microcosm was transferred into a fresh medium. Similar transfers were made for consecutive five weeks. Then, way forward, to find potential culturable members of the community, 1 mL inoculum from the enriched culture media sample was serially diluted in 0.85% (w/v) sterile saline solution and spread on R2A agar (DSM medium No. 830). After incubation for three days at 28 °C, bacterial colonies were isolated and purified with repeated streaking on R2A plates. Strain MAP12^T^ was routinely maintained aerobically on R2A at 28°C. For long-term preservation, it was stored at –80°C in R2A supplemented with 30% (v/v) glycerol.

### Illumina 16S rRNA gene amplicon sequencing for community analysis of enrichment culture

To isolate DNA from the enrichment community, microbial biomass was harvested from 45 mL of the enrichment by centrifuging at 2360 *g* at 4°C for 10 min using a Rotanta 460 R centrifuge (Hettich). Subsequently, DNA was extracted by using the DNeasy Ultraclean Microbial Kit (Qiagen). For evaluation of bacterial community composition the V3 and V4 variable regions of the 16S rRNA gene were amplified by using the universal primer pair suggested by Klindworth et al. (2013) (S-D-Bact-0341-b-S-17 5′-CCTACGGGNGGCWGCAG-3′/S-D-Bact-0785-a-A-215′-GACTACHVGGGTATCTAATCC-3′), containing Illumina adapter overhang. PCR was performed according to the 16S metagenomic sequencing library preparation guide of Illumina using KAPA HiFi HotStart ReadyMix (KAPA Biosystems).

Paired-end fragment reads were sequenced on an Illumina MiSeq sequencer using MiSeq Reagent Kit v3 (600-cycle) by SeqOmics Biotechnology Ltd. (Mórahalom, Hungary). Primary data analysis (base-calling) was conducted with bcl2fastq software (v2.17.1.14, Illumina). Reads were quality and length trimmed in CLC Genomics Workbench Tool 9.5.1 using an error probability of 0.05 (Q13) and a minimum length of 50 nucleotides as a threshold. Then the trimmed sequences were processed using mothur v1.41.1 (Schloss et al., 2009) as recommended by the MiSeq SOP page^2^ (Kozich et al., 2013). Based on the alignment using the SILVA 138 SSU Ref NR 99 database (Quast et al., 2013), sequences were assorted. Detection of chimeras was done with Mothur’s uchime command (Edgar et al., 2011), and the “split.abund” command was also used to remove singleton reads according to Kunin et al. (2010). The 97% similarity threshold level was used to assign Operational taxonomic units (OTUs) as suggested by Tindall et al. (2010) for prokaryotic species delineation.

### Metagenome sequencing and reconstruction of bacterial genomes (genome binning)

Metagenomic DNA quality and integrity was checked using an Agilent 2200 Tapestation system. Paired-end fragment reads (2 × 250 nucleotides) were sequenced using the MiSeq Reagent Kit v2 (500-cycles) on an Illumina MiSeq sequencer. Illumina-artifacts and adapters were removed from raw reads with bbduk^3^ (Bushnell, 2015). Reads were quality controlled and trimmed with sickle version 1.33 (Joshi and Fass, 2011). Quality-controlled reads were assembled using the MetaSPAdes pipeline version 3.15.0 (Nurk et al., 2017). Different binning tools were used to create the final set of bins. Abawaca (v1.00)^4^ was run twice. First including reads with a minimal length of 3000 base pairs (bps) and sequences split after 5000 bps and secondly with minimal scaffold length of 5000 bps and sequences split after 10000 bps. MaxBin2 (v2.2.4) (Wu et al., 2016) was run with both marker sets. All bins created in the four runs were optimized and filtered with DAS Tool (v1.1.2) (Sieber et al., 2018) and hand curated with uBin (Bornemann et al., 2020). The metagenome assembled genome relevant to this study, *Pseudomonas* sp. XYLBin8, can be accessed through NCBI under the BioSample accession number SAMN26818775.

### 16S rRNA gene phylogeny

Genomic DNA from bacterial strains was isolated by using the Ultra- Clean Microbial DNA Kit (Qiagen) following the manufacturers’ instructions. Subsequently, the 16S rRNA gene of the isolates was amplified using the universal bacterial primers 27F and 1492R, than Sanger sequenced by using primers 27F, 338F, 803F and 1492R (Soergel et al., 2012). The reaction parameters of the PCR amplification of 16S rRNA genes and the Sanger sequencing were same as reported earlier (Banerjee et al., 2021). The nearly complete 16S rRNA gene sequences were compared with their closest relatives using the EzTaxon server^5^ to ascertain its taxonomic position (Yoon et al., 2017). Alignment of 16S rRNA gene sequences (ClustalW) and construction of phylogenetic trees were performed by using MEGA (version X) (Kumar et al., 2018) using the neighbor-joining (Saitou and Nei, 1987) and maximum-likelihood (Felsenstein, 1981) methods with Kimura’s two-parameter calculation model and the maximum-parsimony algorithm (Kimura, 1980). Tree topologies and distances were evaluated by bootstrap analysis based on 1000 replicates.

### Genome phylogeny

The whole-genome sequencing of strain MAP12*^T^* was performed by SeqOmics Biotechnology Ltd., Mórahalom, Hungary. *In vitro* fragment libraries were prepared using the NEBNext^®^ Ultra™ II DNA Library Prep Kit for Illumina. Paired-end fragment reads were generated on an Illumina NextSeq sequencer using TG NextSeq^®^ 500/550 High Output Kit v2 (300 cycles). Primary data analysis (base-calling) was carried out with bcl2fastq software (v2.17.1.14, Illumina). Trimmed sequences were *de novo* assembled and scaffolding were performed with SPAdes (v3.13.0) (Nurk et al., 2013). The genome assembly was submitted to NCBI Prokaryotic Genomes Annotation Pipeline (PGAP) v4.5 for automatic annotation (Tatusova et al., 2016). Annotation of the genome was also performed by using the Microbial Genome Annotation & Analysis Platform MicroScope (Vallenet et al., 2020) and using PATRIC 3.5.38 web interfaced pipelines (Wattam et al., 2016). These platforms were also used to determine the general characteristics of the MAP12^T^ genome and to find the presence of genes of interest. Additionally, the putative functions of genes associated with the metabolism of xenobiotics were identified and analyzed using MaGe in combination with the UniProt database^6^ (Bateman, 2019) MetaCyc (Caspi et al., 2014), KEGG (Kanehisa and Goto, 2000) and BLAST searches. Digital DNA–DNA hybridization (dDDH) values among strain MAP12^T^ and related species were determined using the *in silico* Genome-to- Genome Distance Calculator (GGDC^7^) version 2.1 (Meier-Kolthoff et al., 2013). Orthologous average nucleotide identity (OrthoANI) values between strain MAP12^T^ and its closest relatives were estimated using the OAT software (Lee et al., 2016). Reference genomes for comparison purposes were retrieved from the GenBank database.^8^ The phylogenomic tree was constructed *in silico* using a concatenated alignment of 92 core genes with UBCGs software (Na et al., 2018) by applying the FastTree algorithm (Price et al., 2010). The Clusters of Orthologous Groups (COG) functional categories were allocated by digging against the KEGG (Kyoto Encyclopedia of Genes and Genomes) database. The DNA G+C % was estimated from the genomic sequences.

### Physiological analyses

To perform the physiological analysis, reference strains *Pseudomonas sagittaria* DSM 27945^T^ and *Pseudomonas linyingensis* LMG 25967^T^ were obtained from DSMZ–German Collection of Microorganisms and Cell Cultures and BCCM/LGM-Belgian Coordinated Collections of Microorganisms, respectively, and studied under the same laboratory conditions as MAP12^T^. Cell size, shape and arrangement of strain MAP12^T^ were studied by native preparations and by Gram-staining according to Claus (1992). The cell morphology and flagellation type of strain MAP12^T^ was investigated during the exponential growth phase using transmission electron microscopy (H-7100, Hitachi) by applying the shadow-casting technique described by Ohad et al. (1963). According to Barrow and Feltham’s protocol (Barrow and Feltham, 1993) the following physiological and biochemical tests were performed: urease activity; Baird–Parker’s phosphatase activity; production of hydrogen sulpfide from cysteine, indole from tryptophan; hydrolysis of casein, gelatine, aesculin and Tween 80. Catalase activity was determined by bubble production with H_2_O_2_ (3 %, v/v), and oxidase activity was tested using 1 % (w/v) tetramethyl-*p*- phenylenediamineoxalate. Growth at different temperatures from 4 to 50°C (at 4, 10, 20, 28, 37, 45, 50°C) was tested using R2A broth. The optimum pH for growth was estimated using R2A broth, and the pH of the medium was adjusted to 4.0–12.0 (at intervals of 1 pH unit) before autoclaving using citrate/NaH_2_PO_4_ buffer (0.1 M, for pH range 4.0–5.0), phosphate buffer M, for pH range (6.0–7.0) Tris buffer (0.2 M, for pH range 8.0–10.0) and NaOH (5 M, for pH range 10.0–12.0). Salt tolerance was assessed by inoculating the strain into R2A broth supplemented with 0–12 % (w/v) NaCl at 1 % intervals. During pH and salt tolerance tests growth of strain was determined by measuring optical density (OD) at 600 nm. API 50CH, API 20NE and API ZYM strips (bioMerieux) were used to evaluate physiological and biochemical characteristics according to the manufacturer’s instructions. Growth under anaerobic conditions was determined in R2A broth medium with and without the addition of 0.15 % (w/v) KNO_3_ at 28°C. To ensure anaerobic conditions, 100 mL serum bottles (Glasgeratebau Ochs) with 50 mL R2A broth were crimp sealed and purged with nitrogen under sterile conditions as described earlier (Farkas et al., 2015). In addition to that; a growth test was performed using BTEX mixture as carbon source as described earlier (Banerjee et al., 2021). Growth and biomass formation was monitored both by optometric turbidity monitoring and measurement of OD600 value in spectrophotometer after 24 h of incubation.

### Chemotaxonomic characterization

Whole-cell fatty acid, respiratory quinones and polar lipids analysis were performed by the Identification Service of DSMZ (Braunschweig, Germany). To analyze fatty acid methyl esters, strain MAP12^T^ and the reference strains were cultivated on R2A agar at 28°C. Sufficient cells of comparable physiological age could be harvested from the third quadrant of the plates. Fatty acid methyl esters analysis was performed following the instructions of the Sherlock Microbial Identification System version 6.4 (midi) and identified using the TSBA6 database (v.6.21). The polar lipids and menaquinones were extracted based on the modified procedure of Bligh and Dyer (1959). Accordingly, polar lipids were extracted from freeze dried cell material using a choroform:methanol:0.3% aqueous NaCl mixture, polar lipids were recovered into the chloroform phase. Subsequently, polar lipids were separated by two dimensional silica gel thin layer chromatography. The first direction was developed in chloroform:methanol:water, and the second in chloroform:methanol:acetic acid:water. Total lipid material was detected using molybdatophosphoric acid and specific functional groups were detected using spray reagents specific for defined functional groups. The quinones were analyzed by LDC Analytical (Thermo separation Products) HPLC apparatus fitted with a reverse-phase column (Macherey-Nagel; 2.125 mm, 3 μm, RP18) using methanol:heptane 9: 1 (v/v) as eluant.

### Determination of aerobic and microaerobic benzene, toluene, ethylbenzene and xylene degradation potential of strain MAP12^T^

BTEX (benzene, toluene, ethylbenzene, *o*-, *m-* and *p*-xylene) degradation ability of strain MAP12^T^ was assessed using individual BTEX compounds as the sole source of carbon and energy in microcosm experiment under strict aerobic (DO conc. 8 mg/L) and microaerobic conditions (DO conc. 0.5 mg/L) by GC-MS. Measurements were carried out in triplicates, along with triplicates of non-inoculated samples, which served as the abiotic negative controls. The experiment was conducted in 100 mL crimped serum bottles containing 50 mL MSV medium (Révész et al., 2020) supplemented with different BTEX compounds as a carbon source at 5 mg/L concentration and kept aside for 24 h to provide time for the abiotic solution to reach the equilibrium state. Thereafter, investigational 100 μl 24 h old bacterial inoculum (OD600 0.5) was added to the serum bottles. Additionally, negative serum bottles are kept as it is, as uninoculated. Then, all of the bottles (both inoculated and negative controls) were kept for incubation at 28°C and 150 r.p.m in a rotary shaker incubator. Biodegradation of BTEX compounds was measured at a regular interval of 24 h by headspace analysis in GC-MS. Microaerobic condition in the microcosms was set as described above. In short, microcosms were sparged aseptically with N_2_:CO_2_ (80:20) gas mixture to remove oxygen content and then desired amount of sterile air was injected into the bottles to achieve desired oxygen concentration in the microcosms. BTEX concentration during the experiments was determined by GC-MS from the headspace. Sampling was performed using an SPME polydimethylsiloxane fiber assembly (Supelco), and the analysis was executed using Trace 1300 gas chromatograph coupled to ISQ Single Quadrupole mass spectrometer (ThermoFisher Scientific). During the investigation, injector and detector temperatures were kept at, respectively, 200°C and 250°C. The oven temperature was set to 40°C for 3 min, later ramped at a rate of 20°C min^–1^ to 190°C and lastly held at that temperature for 1 min. As carrier gas, Helium 5.0 was used at a flow rate of 1.2 ml min^–1^. For separation (30 m × 0.25 mm × 0.25 μm, Sigma-Aldrich, Supelco) SLB-5 ms fused silica capillary column was used. The mass spectrometer was operated in full scan mode.

To observe the growth of strain MAP12^T^ during microaerobic degradation of BTEX-compounds batch experiment was conducted for 96 h, similarly as it was described above, with minor modifications. The initial concentration of the individual BTEX-compounds was 1 mM to support biomass formation. At regular intervals the liquid phase was sampled by a gas-tight glass syringe and OD600 was measured by using a NanoDrop One Microvolume UV-Vis spectrophotometer (ThermoFisher Scientific). At the same time, BTEX-concentration was measured in the headspace of the bottles similarly as described above. Uninoculated bottles were used as negative controls during the experiment. All batch experiments were performed in triplicate.

## Results and discussion

### Enrichment of microaerobic xylene-degraders: Isolation and phylogenetic analysis of strain MAP12^T^

**FIGURE 1:**
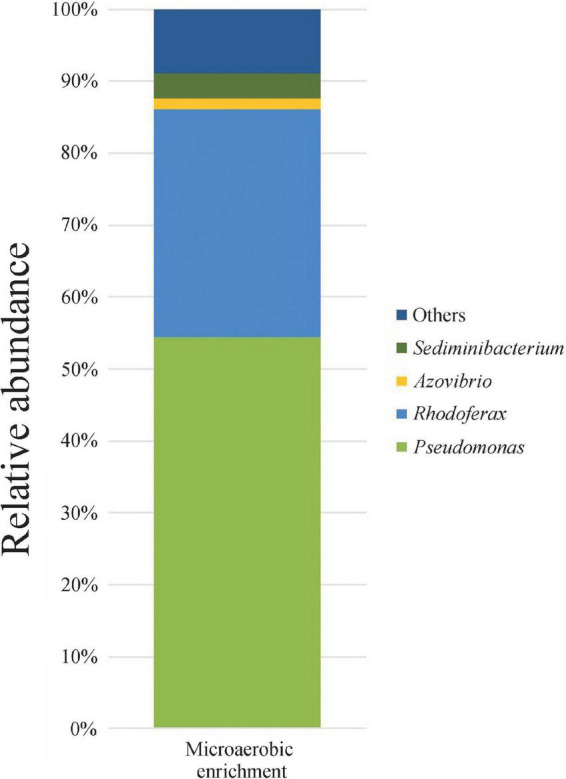
Genus-level bacterial community structure of microaerobic xylene-degrading enrichment as revealed by Illumina paired-end 16S rRNA gene amplicon sequencing. Only taxa contributing more than 1% abundance were depicted.

In the enrichments, rapid and complete degradation of all three isomers of xylene was observed. Less than 48 h of incubation were needed for the complete degradation of *m*-, and *p*-xylene, while the degradation time for *o*-xylene was extended to 96 h (data not shown). For strain isolation, one of the enrichments (MIC3) was chosen. 16S rRNA gene amplicon sequencing of this enrichment revealed that the structure of the enriched bacterial community was rather simple, since only 33 OTUs were detected. The bacterial community was overwhelmingly dominated by members of the genus *Pseudomonas* (54.4% relative abundance), followed by *Rhodoferax* (31.7%) and *Sediminibacterium* (3.6%) (Figure 1). Most of the isolates gained from this enrichment belonged to the genus *Pseudomonas*, while members of the genera *Rhodoferax* and *Sediminibacterium* were not isolated (data not shown).

**FIGURE 2:**
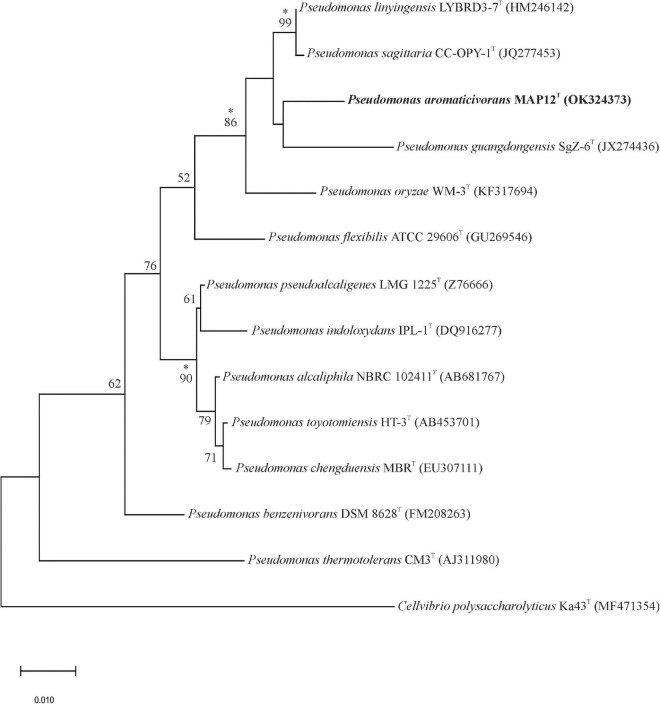
Maximum-likelihood tree based on 16S rRNA gene sequences showing the phylogenetic relationships between strain MAP12^T^ and related taxa. Bootstrap values are shown as percentages of 1000 replicates. Branches signed with an asterisk occurred with every tree-making algorithm used in the study. *Cellvibrio polysaccharolyticus* Ka43^T^ was used to root the tree. Bar, 0.01 substitution per nucleotide position.

The 16S rRNA gene sequence analysis of the isolates showed that a strain, designated as MAP12^T^ formed a distinct, undescribed phylogenetic lineage of the genus *Pseudomonas*. Strain MAP12^T^ exhibited the closest relatedness to *Pseudomonas linyingensis* LYBRD3-7^T^ with 98.42% 16S rRNA gene sequence similarity followed *by Pseudomonas sagittaria* JCM 18195^T^ (98.29% similarity) and *Pseudomonas alcaliphila* JCM 10630^T^ (98.08% similarity). The 16S rRNA gene sequence similarities between strain MAP12^T^ and other members of the genus *Pseudomonas* were below 98%. The maximum-likelihood phylogenetic tree based on the 16S rRNA sequences (Figure 2) showed that strain MAP12^T^ formed a separate lineage within a cluster containing *Pseudomonas sagittaria* CC-OPY-1^T^, *Pseudomonas linyingensis* LYBRD3-7^T^ and *Pseudomonas guangdongensis* SgZ-6^T^. The separate lineage formed in both neighbor-joining and maximum-likelihood trees strongly supported the identification of strain MAP12^T^ as a novel member in the genus *Pseudomonas* (Supplementary Figures 1, 2). Based on the abovementioned results, *P. sagittaria and P linyingensis* were selected as reference type strains for further side-by-side analyses with strain MAP12^T^.

### Phylogenomic analyses

**FIGURE 3:**
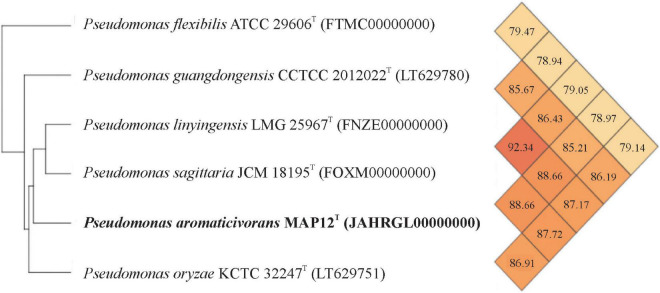
Heatmap generated with OrthoANI values between strain MAP12^T^ and other closely related type strains of the genus *Pseudomonas*.

**FIGURE 4:**
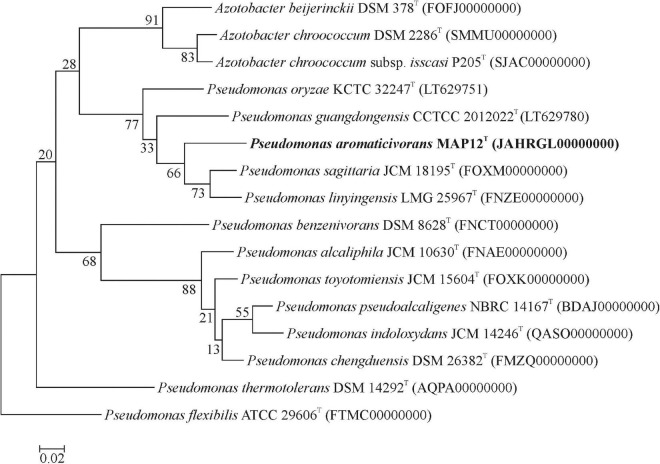
Phylogenomic tree constructed using UBCGs (concatenated alignment of 92 core genes). For inferring the tree the FastTree algorithm was used. Bar, 0.05 substitution per nucleotide position.

The draft assembly of the whole-genome shotgun sequence has been deposited at DDBJ/ENA/GenBank under the accession JAHRGL000000000. The assembled genome consists of a single circular chromosome with 89 contigs and a total length of 4,365,611 bp. The MAP12^T^ genome has an average G+C content of 65.8 % and contains 3987 protein-coding sequences. To evaluate the genomic relatedness of MAP12^T^ to its closest relatives, the genome sequences of *Pseudomonas sagittaria* JCM 18195^T^ (FOXM01000000), *Pseudomonas linyingensis* LMG 25967^T^ (FNZE01000000), *Pseudomonas oryzae* KCTC 32247^T^ (NZ_LT629751.1), *Pseudomonas guangdongensis* CCTCC AB 2012022^T^ (NZ_LT629780.1) and *Pseudomonas flexibilis* ATCC 29606^T^ (FTMC01000000) were downloaded from NCBI and used for determining OrthoANI and dDDH values. The OrthoANI values between strain MAP12^T^ and phylogenetically closest neighbors were between 78 and 88% (Figure 3), which is much lower than the threshold value of 95-96%, recommended for species level delineation (Meier-Kolthoff et al., 2013). Regarding dDDH analysis, strain MAP12^T^ showed the highest dDDH value with *P. sagittaria* JCM 18195^T^ (35.8%), followed by *P. linyingensis* LMG 25967^T^ (35.6%) and *P. guangdongensis* CCTCC AB 2012022^T^ (29.4%). Consequently, both the OrthoANI and dDDH values supported the species level delineation of strain MAP12^T^. Furthermore, the phylogenomic tree constructed using UBCGs (concatenated alignment of 92 core genes) also proved that strain MAP12^T^ was a novel member of the genus *Pseudomonas* (Figure 4).

Genome-resolved metagenomics analysis of enrichment MIC3 revealed the abundance of strain MAP12^T^ in the enrichment community. As a result of genome binning, twelve high quality metagenome-assembled genomes (MAGs) were acquired, and two of them could be affiliated to the genus *Pseudomonas*. Although one of the *Pseudomonas* MAGs, designated as XYLBin8 (accessible at NCBI under the BioSample number SAMN26818775) represented only a partial genome (1.9 Mb large sequence, 23 contigs), it could be affiliated with the *P. sagittaria*/*linyingensis* lineage. The OrthoANI analysis between this MAG and the whole genome sequence of strain MAP12^T^ yielded a value of 99.94% on the overlapping parts, indicating that the genome of strain MAP12^T^ could also be partly reconstructed from the metagenome sequence data. By mapping sequence data of the XYLBin8 MAG to trimmed, quality-controlled reads the relative abundance of XYLBin8 was found to be 8.66% in the metagenome (mean coverage of 95.39x). Consequently, it can be assumed that strain MAP12^T^ was an abundant member of the enrichment community and could be a key microaerobic xylene degrader.

### Physiological analysis

**FIGURE 5:**
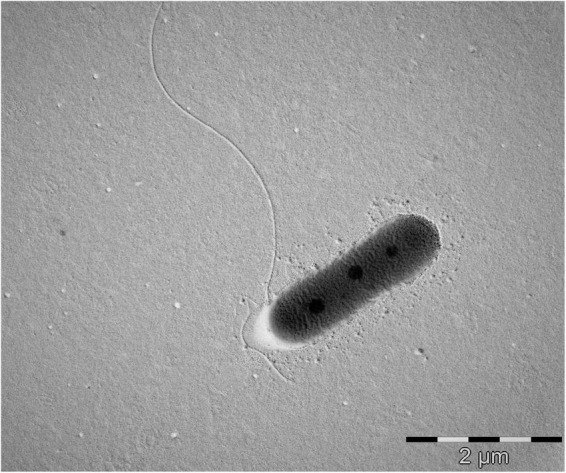
Transmission electron microscopic photograph showing cell morphology and presence of flagella in strain MAP12^T^. Bar = 2 μm.

The cells of strain MAP12^T^ are rod-shaped, Gram-stain-negative, aerobic and motile with a polar flagellum. The cells were approximately 2.2–2.5 μm in length and 0.6–0.8 μm in diameter (Figure 5 and Supplementary Figure 3). Strain MAP12^T^ formed white, glossy, opaque, and circular colonies after 24 h of incubation on R2A at 28°C. The strain was negative for the indole test. Strain MAP12^T^ grew well, but the reference strains were unable to grow at 4°C. MAP12^T^ can grow at a wide temperature range of 4–45°C, at a pH range from 5.0 to 12.0, and can tolerate up to 6 % NaCl (Table 1). Moreover, strain MAP 12^T^ grew better in saline (1–4% NaCl) than in the salt-free control. Additionally, MAP12^T^ was unable to hydrolyze Tween 80 and positive for catalase and oxidase test. In API ZYM tests, MAP12^T^ showed positive enzymatic activity for esterase (C4), esterase lipase (C8), leucine arylamidase, acid phosphatase, naphthol-AS-BI-phosphohydrolase and weakly positive results were detected for leucine arylamidase and trypsin. For API 20NE, strain MAP12^T^ showed positive results for urease activity and esculin ferric citrate (β-glucosidase) hydrolysis. Whereas it showed negative reactions for aerobic NO_3_ reduction. Moreover, MAP12^T^ exhibited assimilation of D-glucose, D-mannose, adipic acid and malic acid. The results of API50 CH tests showed that strain MAP12^T^ could metabolize substrates like glycerol, D-fructose, inositol, esculin ferric citrate and D-arabitol. In the biochemical tests, the strain showed positive results for indole production and MR test. In the anaerobic growth monitoring experiment, there was no growth observed after incubation, proving that nitrate does not support the growth of MAP12^T^ in the absence of oxygen. The detailed comparative characteristics are given in Table 1 with phylogenetically closest species of the genus *Pseudomonas*.

**TABLE 1:** Major phenotypic and biochemical characteristics of strain MAP12^T^ and other closely related type strains Strains: 1, MAP12^T^; 2, *Pseudomonas sagittaria* DSM 27945^T^; 3, *Pseudomonas linyingensis* LMG 25967^T^.

Characteristics	1	2	3
Temperature range for growth (°C)	4–45	4–45	4–45
pH range for growth	5–12	5.5–12	5–12
NaCl tolearnce (%, w/v)	1–5	1–4	1–4
**Enzyme activities (API ZYM):**			
Alkaline phosphatase	–	+	+
Esterase (C 4)	+	+	+
Esterase lipase (C 8)	+	+	+
Leucine arylamidase	+	+	+
Valine arylamidase	w+	+	-
Cystine arylamidase	-	+	-
Trypsin	w+	+	+
Acid phosphatase	+	+	+
Naphthol-AS-BI-phosphohydrolase	+	+	+
α- galactosidase	–	–	+
β- galactosidase	–	–	+
β- glucunoridase	–	–	+
*α-* glucosidase	–	+	+
*β-* glucosidase	–	+	+
N-acetyl-β-glucosaminidase	–	w+	+
α- mannosidase	–	w+	+
α- fucosidase	–	w+	+
**Assimilation of (API 20NE):**			
Urea (urease)	+	+	+
Esculin ferric citrate (β-glucosidase) hydrolysis	+	+	+
Gelatin (protease) hydrolisys	–	–	–
4-nitrophenyl-βD-galactopyranoside (β-galactosidase)	+	+	+
D-glucose (assimiliation)	+	–	+
D-mannose	+	–	–
Adipic acid	+	–	–
Maltic acid	+	+	+
Trisodium citrate	–	+	–
**Acid production from (API 50 CHB/E)**:			
Glycerol	+	–	–
D-fructose	+	+	-
Inositol	+	–	+
D-mannitol	–	–	+
D-sorbitol	–	+	–
Esculin ferric citrate	+	–	+
D-arabitol	+	–	–

+, positive/utilized; w+, weak positive; –, negative/not utilized. All data were obtained in this study.

### Microaerobic and aerobic benzene, toluene, ethylbenzene and xylene degradation analysis of strain MAP12^T^

**FIGURE 6:**
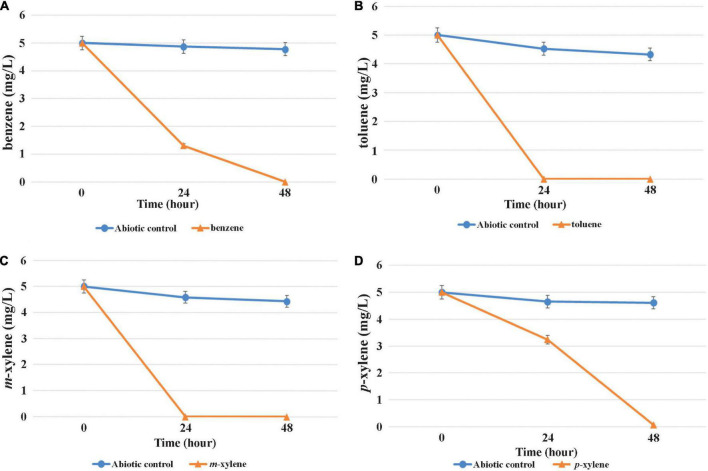
Microaerobic degradation of BTEX compounds [**(A)**: benzene, **(B)**: toluene, **(C)**: *m*-xylene, **(D)**: *p*-xylene] by strain MAP12^T^. Concentrations were determined by GC–MS analysis as described in the main text. The averages of triplicate experiments ± standard errors of the means, indicated by error bars, are shown.

The capability of MAP12^T^ to grow and degrade BTEX compounds under both aerobic and oxygen-limited conditions was evaluated using individual BTEX compounds as the sole source of carbon and energy to understand the difference in BTEX biodegradation pattern influenced by oxygen availability. It turned out that the strain was able to degrade benzene, toluene, *m*- and *p*-xylene effectively even under microaerobic conditions (Figure 6). In contrast, data obtained from the degradation experiment revealed that it was unable to degrade *o*-xylene and ethylbenzene. Complete microaerobic degradation of 5 mg/L of toluene and *m*-xylene took place within 24 h and benzene within 24 h to 48 h. Results of the aerobic degradation experiment disclosed that MAP12^T^ can degrade the same BTEX compounds within a similar time frame (data not shown). Results of the microaerobic growth test (in which the individual BTEX-compounds were provided in a concentration of 1 mM) indicated that MAP12^T^ can use the investigated BTEX-compounds as sole growth substrates since notable biomass formation was observed during microaerobic degradation (Supplementary Figure 4). It was also observable, that toluene was the preferred growth substrate under microaerobic conditions, while the slowest biomass formation and degradation was observed in case of benzene. According to the literature, though there are several documented members of genus *Pseudomonas*, which can degrade BTEX compounds aerobically, but this is the first report on a *Pseudomonas* strain that could degrade benzene and xylene isomers, as a pure culture under microaerobic conditions. This finding is also supported by the presence of a subfamily I.2.C type C23O gene, which is known to play a critical role in the degradation of BTEX compounds under microaerobic conditions (Táncsics et al., 2012).

### Genomic overview of strain MAP12^T^ with emphasis on aromatic hydrocarbon degradation

The detailed profiling of clusters of orthologous groups (COG) provides in-depth details into the genomic potential of this isolate regarding its ability of degradation and survival in hydrocarbon-contaminated complex habitats (Supplementary Figure 5). From the perspective of this study, special emphasis was put on those gene clusters, which could have a role in the biodegradtion of petroleum hydrocarbons. The presence of five catechol 2,3-dioxygenase (C23O) genes (Sequence Locus Tags KRX52_06295, KRX52_06950, KRX52_07180, KRX52_11010, KRX52_11075) in the genome is presumably the reason behind the effective BTEX degradation capability of this organism. Moreover, one of the C23O genes (locus tag KRX52_11075) encoded a subfamily I.2.C-type extradiol dioxygenase, which ring–cleavage enzymes are known to function under hypoxic conditions, thus enable aerobic ring-cleavage under microaerobic conditions as well (Kukor and Olsen, 1996). Genome mining also indicated the presence of multiple genes for the complete lower *meta*-cleavage pathway, essential for the introduction of ring-cleavage intermediates to the TCA cycle. Automatic annotation results of the MaGe identified the presence of genes encoding enzymes involved in the utilization of various aromatic compounds in strain MAP12^T^ as a source of carbon and energy (Supplementary Figure 6). In general, biodegradation of xylene is initiated by the oxidation of a methyl substituent to form methylbenzyl alcohol by xylene monooxygenase (XylM) (Yu et al., 2001; Jung et al., 2005; Cao et al., 2009). In this xylene monooxygenase pathway, the methylbenzyl alcohol is subsequently converted to methylbenzaldehyde, methylbenzoate and 1,2-dihydroxy-methylcyclohexane-3,5-dienecarboxylate by benzylalcohol dehydrogenase (XylB), benzaldehyde dehydrogenase (XylC) and benzoate 1,2-dioxygenase (XylX), respectively, and later with the help of catechol 2,3-dioxygenase (XylE) as a ring cleavage enzyme, and lower *meta*-cleavage pathway enzymes converted to pyruvate through a series of enzymatic reactions. The *xylECMABN* genes were found in the genome of strain MAP12^T^ as part of a partial (only upper TOL pathway genes containing) gene cluster (between locus tags KRX52_11065 and KRX52_11120). Moreover, this *xyl* gene cluster was flanked by mobile genetic elements (IS3 family transposases) and the subfamily I.2.C *C23O* gene (encoding the XylE enzyme) was part of this cluster. Consequently, it can be suggested, that this cluster plays crucial role in the microaerobic degradation of toluene, *p*- and *m*-xylene in case of strain MAP12^T^. However, it is well-known that the presence of the subfamily I.2.C-type *C23O* gene is not the only requirement for the ability of microaerobic degradation of monoaromatic hydrocabons. Prior to the ring cleavage, the aromatic ring has to be activated by a mono- or dioxygenase enzyme, which also has to function under microaerobic conditions. Here a benzoate/toluate 1,2-dioxygenase (XylX) plays this role. Unfortunately, there is no information on the activity of these enzymes under microaerobic conditions. In general, it was suggested by Martínez-Lavanchy et al. (2015) that ring-monooxygenation is the preferred pathway under microaerobic conditions, rather than dioxygenation. On the other hand, it was observed in case of *Thauera* sp. DNT-1 and *Zoogloea oleivorans* Buc^T^ that a toluene dioxygenase-like enzyme was responsible for the ring-activation under microaerobic conditions (Shinoda et al., 2004; Táncsics et al., 2020). Thus, transcriptomic analysis will be needed to clearly resolve the microaerobic degradation pathway of aromatic hydrocarbons in strain MAP12^T^.

Genes encoding enzymes that needed for utilizing ethylbenzene were not found in the genome. The absence of genes such as ethylbenzene dioxygenase or naphthalene dioxygenase explains its inability to consume ethylbenzene as a growth substrate. Furthermore, the reason behind the benzene-degrading potential of strain MAP12^T^ could be due to the presence of a phenol-degradation gene cluster in its genome encoding for a multicomponent phenol-hydroxylase system together with a complete *meta*-cleavage pathway. In addition, the existence of cold-shock genes *cspA* and *cspC* in the genome of strain MAP12^T^ probably explains its ability to grow at low temperatures. These aforementioned genome features make this strain a potential bioremediation candidate for contaminated subsurface, especially for groundwater reserves where the temperature is low, and oxygen availability is limited.

### Chemotaxonomic characterization

The major fatty acids (>10%) of strain MAP12^T^ were summed feature 3 (C_16:1_ω6c and/or C_16:1_ω7c), C_16:0_, and summed feature 8 (C_18:1_ω6c and/or C_18:1_ω7c), which are generally present in species of the genus *Pseudomonas*. The major fatty acid composition supported the affiliation of MAP12^T^ with the genus *Pseudomonas*, although some quantitative differences from the reference strains were observed. The details of fatty acid profiles of MAP12^T^ and other closely related species are given in Table 2. The major respiratory quinones were ubiquinone-9 (Q-9) (86.7%) and ubiquinone-8 (Q-8) (13.3%) which is compatible with other species of the genus *Pseudomonas*. The major polar lipids were diphosphatidylglycerol, phosphatidylglycerol, and phosphatidylethanolamine (Supplementary Figure 7). Results of the chemotaxonomic analysis are in agreement with data published previously for species of the genus *Pseudomonas* (Cámara et al., 2007; Stolz et al., 2007; Romanenko et al., 2008).

**TABLE 2:** Cellular fatty acid compositions of strain MAP12^T^ and related species.

Fatty acid	1	2	3
**Saturated**			
C_12:0_	7.6	7.3	7.3
C_16:0_	27.6	22.8	22.0
**Hydroxy**			
C_10:0_ 3OH	4.0	4.0	3.9
C_12:0_ 3OH	3.6	3.5	3.7
**Cyclic**			
C_17:0_ cyclo	3.7	3.5	3.6
C_19:0_ cyclo *ω8*c	1.3	1.2	1.9
**Summed feature** *			
3	34.7	33.7	33.9
8	15.9	22.9	22.0

Taxa: 1, strain MAP12^T^; 2, Peudomonas sagittaria DSM 27945^T^; 3, Pseudomonas linyingensis LMG 25967^T^; Data are expressed as percentages of total fatty acids. Fatty acids which were lower than 1.0% in all strains are not shown. All data are from the present study. * Summed Features are fatty acids that cannot be resolved reliably from another fatty acid using the chromatographic conditions chosen. The MIDI system groups these fatty acids together as one feature with a single percentage of the total. Summed feature 3, C_16:1_ω6c and/or C_16:1_ω7c; summed feature 8, C_18:1_ω6c and/or C_18:1_ω7c.

### Description of *Pseudomonas aromaticivorans* sp. nov.

*Pseudomonas aromaticivorans* (a. ro. ma. ti. ci. vo’rans. L. masc. adj. *aromaticus* fragrant; L. pres. part. *Vorans* devouring; N.L. part. adj. *aromaticivorans* devouring aromatic compounds). Cells are Gram stain-negative, aerobic, rod-shaped, approximately 2.2-2.5 μm in length and 0.6–0.8 μm in diameter, and motile due to the presence of a single polar flagellum. Can grow at temperatures ranging from 4 to 45°C, in the presence of 0–6% (w/v) NaCl and at pH values of 5–12. Colonies are colorless, circular after incubation for 2–3 days on R2A agar at 28 °C The optimal growth temperature is 28°C, and the optimal pH is 7.0, prefers the presence of 1–4% NaCl. It is positive for catalase and oxidase. Furthermore, it shows positive enzymatic activity for esterase (C4), esterase lipase (C8), leucine arylamidase, acid phosphatase, Naphthol-AS-BI-phosphohydrolase, and weakly positive results for leucine arylamidase and trypsin. Positive for urease activity, esculin ferric citrate (β-glucosidase) hydrolysis and assimilation of D-glucose, D-mannose, adipic acid and malic acid, can assimilate glycerol, D-fructose, inositol, esculin ferric citrate and D-arabitol. The major cellular fatty acids are summed feature 3 (C_16:1_ω6c and/or C_16:1_ω7c), C_16:0_ and summed feature 8 (C_18:1_ω6c and/or C_18:1_ω7c). Ubiquinone-9 (Q-9) is the major respiratory quinone. The major polar lipids are - diphosphatidylglycerol, phosphatidylglycerol and phosphatidylethanolamine. It is able to degrade benzene, toluene, *m*- and *p*-xylene, but not ethylbenzene and *o*-xylene. The DNA G+C content is 65.80%. The type strain, MAP12^T^ (=LMG 32466, =NCAIM B.02668), was isolated from microaerobic xylene-degrading enrichment culture inoculated with contaminated groundwater sample of the Siklos BTEX contaminated site (Hungary).

## Data availability statement

The datasets presented in this study can be found in online repositories. The names of the repository/repositories and accession number(s) can be found below: https://www.ncbi.nlm.nih.gov/genbank/ (OK324373) and https://www.ncbi.nlm.nih.gov/ (JAHRGL000000000.1, PRJNA745543, and PRJNA818156).

## Author contributions

SB: enrichment experiments and strain isolation (lead), phylogenetic and genomic analyses (lead), writing — original draft (equal), and review and editing of final manuscript (equal). AB: enrichment experiments and strain isolation (equal). ET: physiological analysis of strain MAP12^T^ and reference strains (lead), and review and editing of final manuscript (equal). AS: genome binning (lead) and review and editing of final manuscript (equal). BK: review and editing of final manuscript (equal). KB: transmission electron microscopy (lead). AT: conceptualization (lead), writing – original draft (equal), and review and editing of final manuscript (equal). All authors read and approved the final version.

## Footnotes

1
https://www.bacterio.net/index.html

2
http://www.mothur.org/wiki/MiSeq_SOP

3
https://sourceforge.net/projects/bbmap/

4
https://github.com/CK7/abawaca

5
www.ezbiocloud.net

6
http://www.uniprot.org/

7
https://ggdc.dsmz.de/

8
www.ncbi.nlm.nih.gov/genbank
