# Supplementary material for: Isolation of Pseudomonas aromaticivorans sp. nov from a hydrocarbon-contaminated groundwater capable of degrading benzene-, toluene-, m- and p-xylene under microaerobic conditions
Source: Front Microbiol. 2022 Sep 20;13:929128. doi: 10.3389/fmicb.2022.929128 (PMC9530055; doi:10.3389/fmicb.2022.929128)
Supplement: Supplementary file 1 [file Data_Sheet_1.PDF]

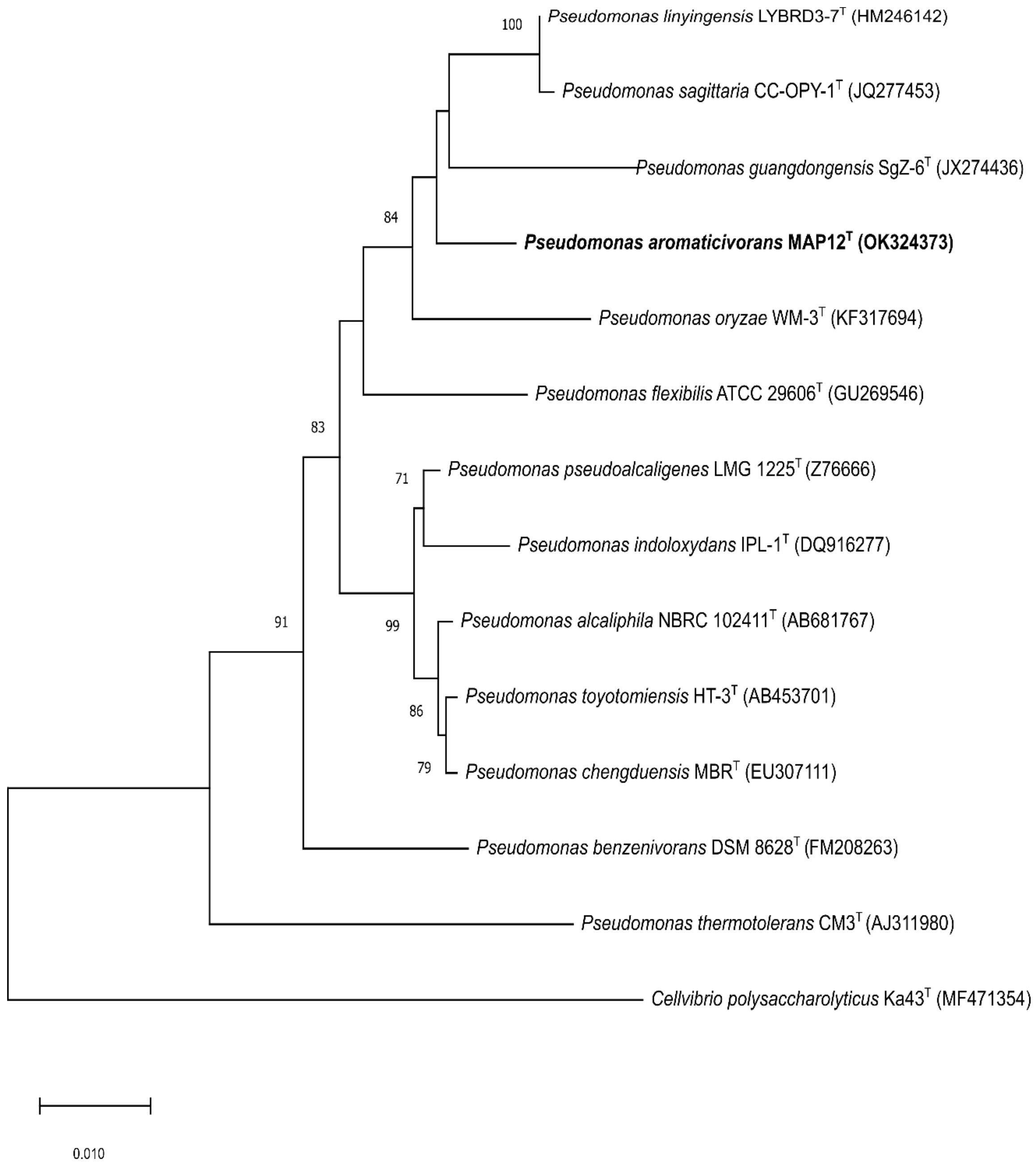


**Supplementary Figure 1**. Neighbor-joining tree based on 16S rRNA gene sequences showing the phylogenetic relationships between strain MAP12$^T$ and related taxa. Bootstrap values (only >50) are shown as percentages of 1000 replicates. *Cellvibrio polysaccharolyticus* Ka43$^T$ was used to root the tree. Bar, 0.01 substitution per nucleotide position.

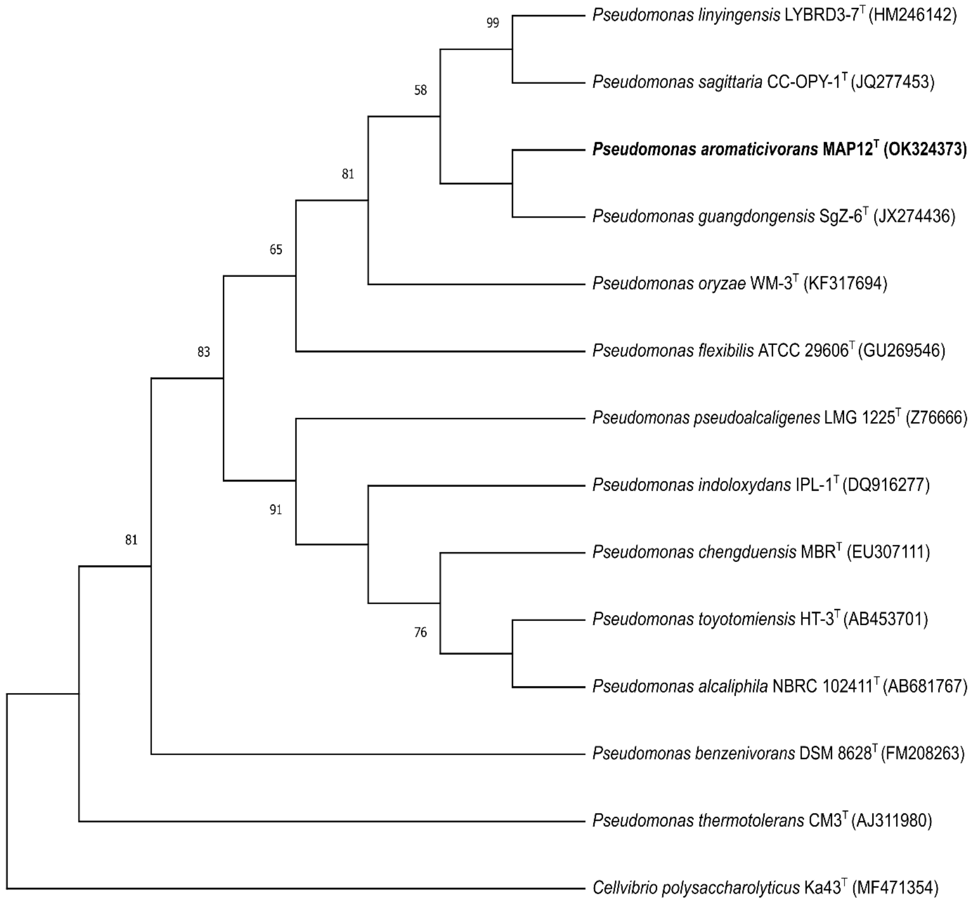


**Supplementary Figure 2**. Maximum parsimony tree based on 16S rRNA gene sequences showing the phylogenetic relationships between strain MAP12$^T$ and related taxa. Bootstrap values (only >50) are shown as percentages of 1000 replicates. *Cellvibrio polysaccharolyticus* Ka43$^T$ was used to root the tree.

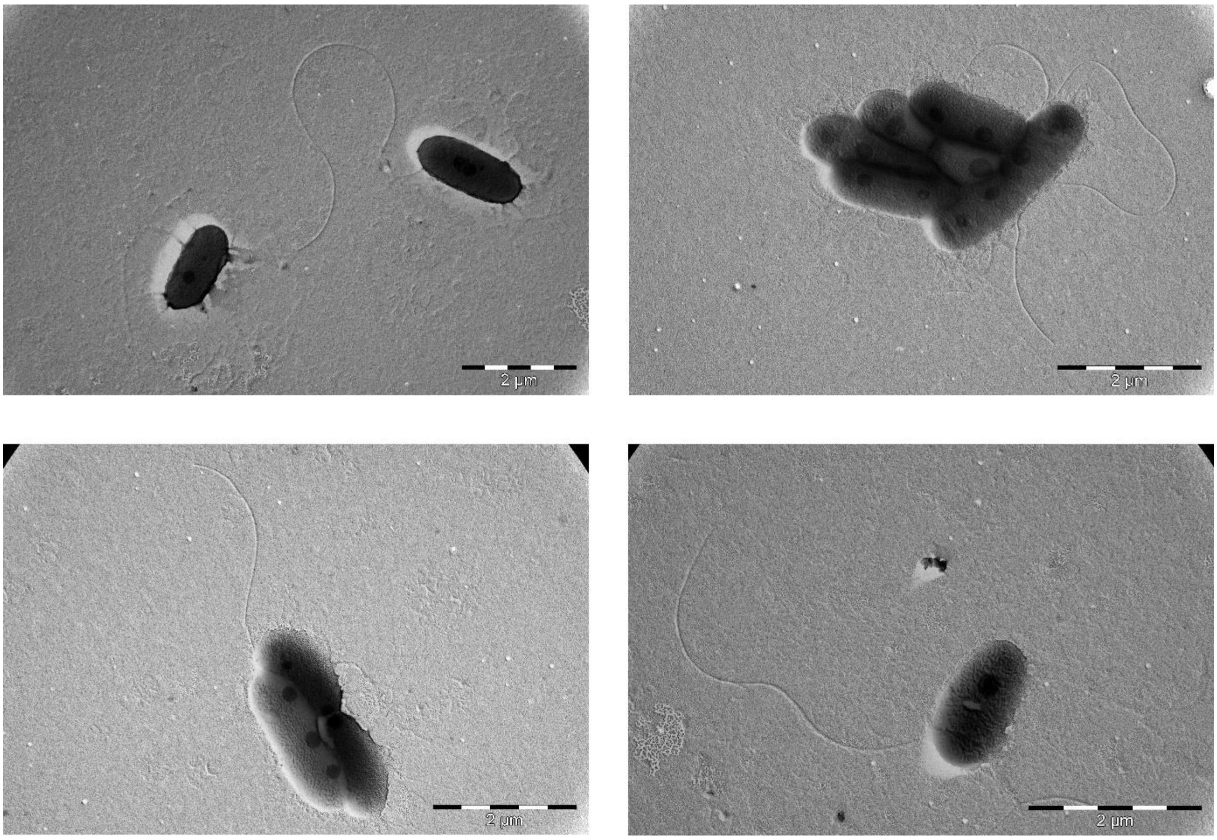


**Supplementary Figure 3.** Transmission electron microscopic photographs showing cell morphology and presence of flagella in case of strain MAP12$^{T}$. Bar = 2 μm.

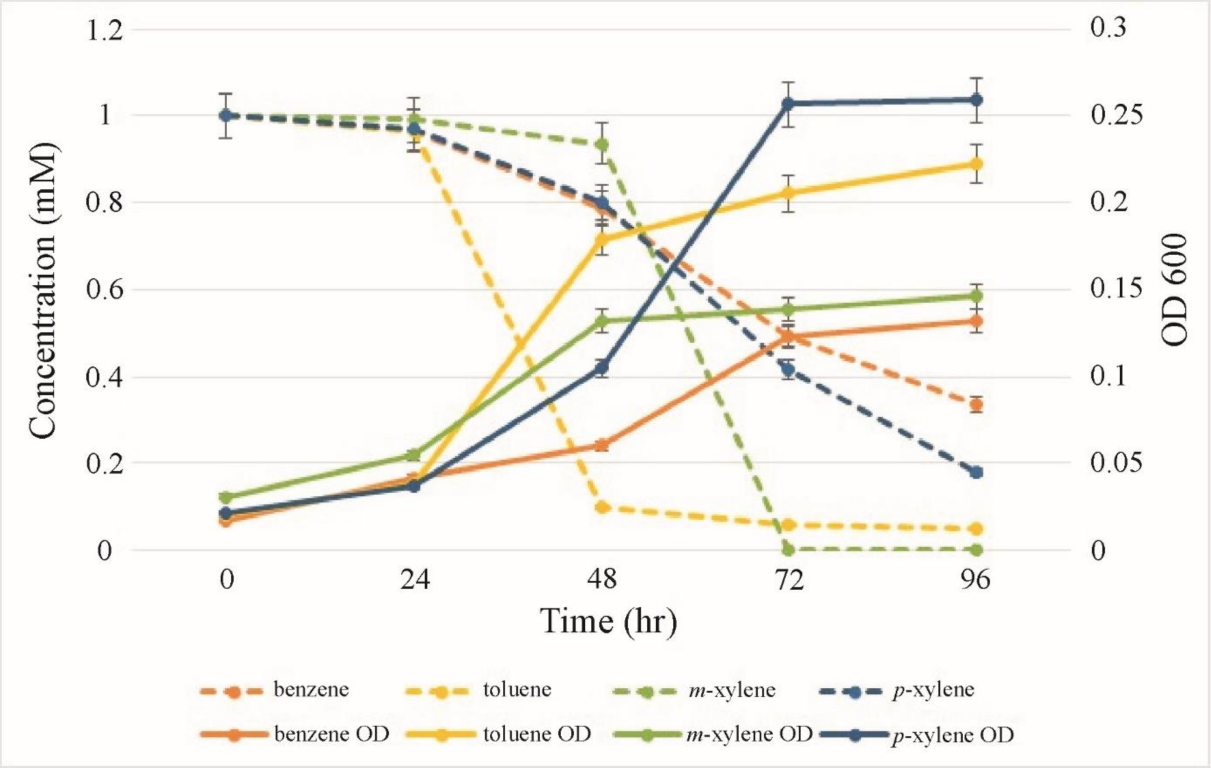


**Supplementary Figure 4.** Growth curves of strain MAP12[T] during the microaerobic degradation of either benzene, toluene, *m*- or *p*-xylene. Solid lines represent absorbance values recorded at 600 nm, while dashed lines represent aromatic hydrocarbon concentration in mM. The averages of triplicate experiments ± standard errors of the means, indicated by error bars, are shown.

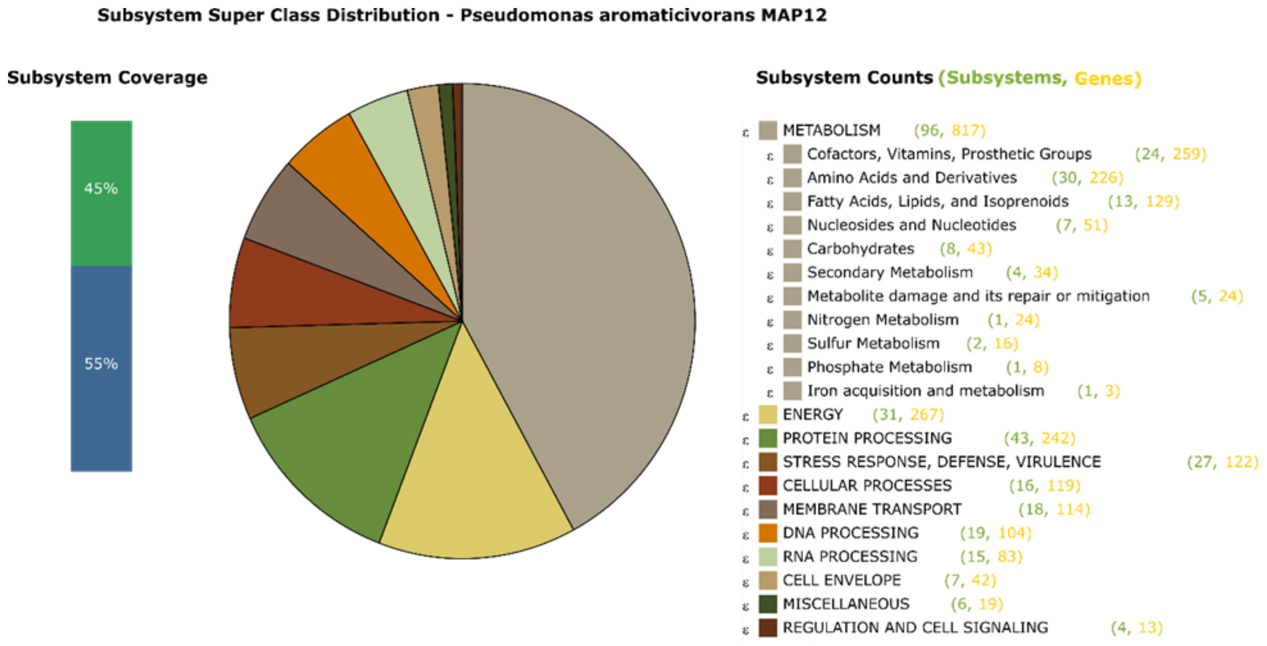


**Supplementary Figure 5.** Subsystem coverage and category distribution of whole genome. The pie chart indicates the counts of each subsystem feature and the subsystem coverage. The number of genes in each subsystem category was shown in brackets.

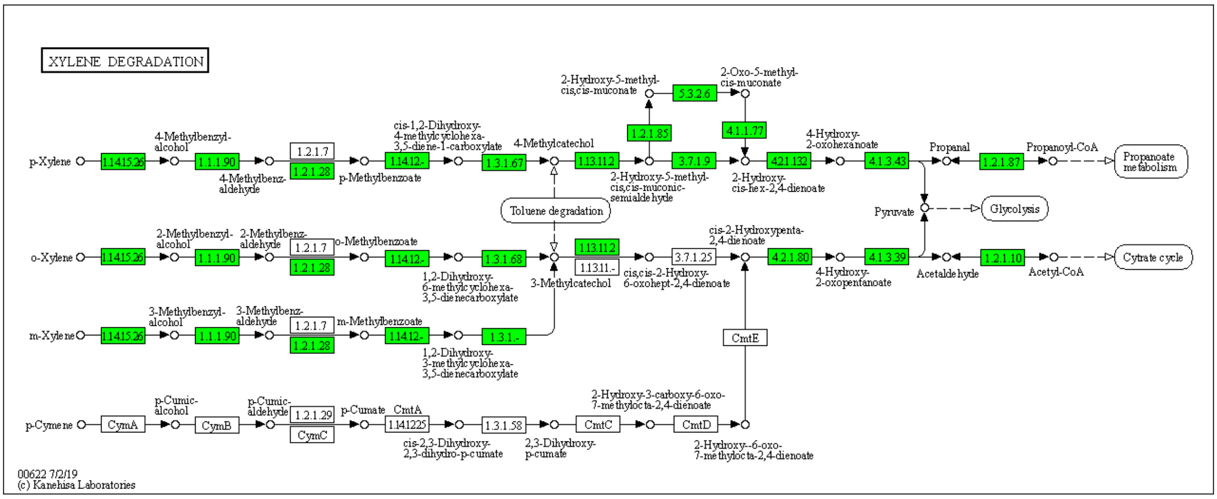


**Supplementary Figure 6**. Automated annotation of xylene degradation pathway of strain MAP12$^T$ by MaGe Microscope platform.

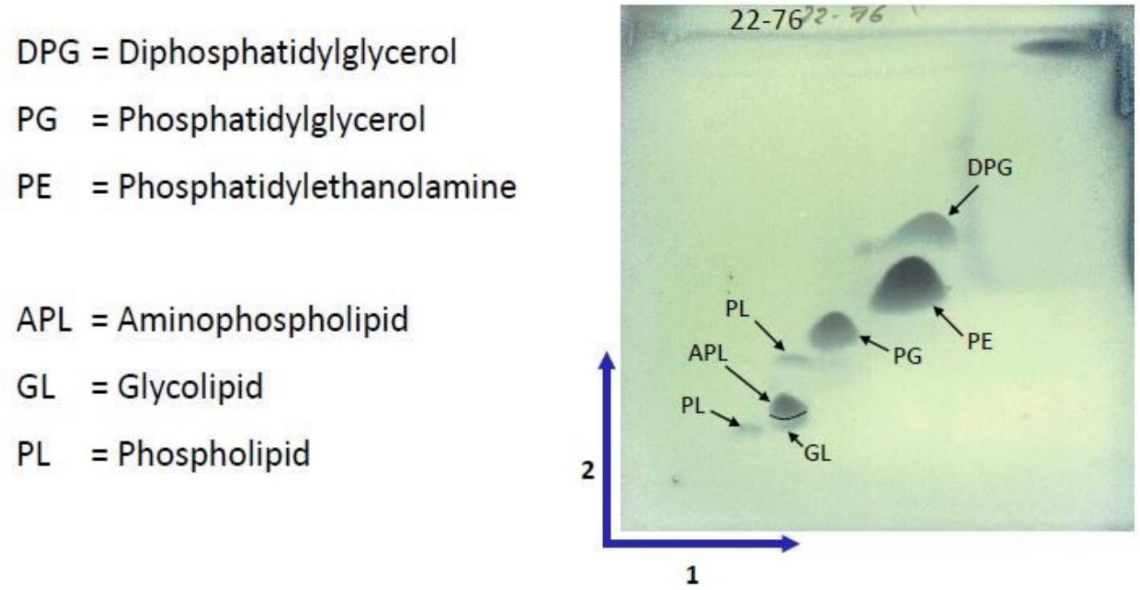


**Supplementary Figure 7**. Two-dimensional TLC of the total polar lipids of strain *Pseudomonas aromaticivorans* MAP12$^{T}$
